# Making Space for Midwifery in a Hospital: Exploring the Built Birth Environment of Canada’s First Alongside Midwifery Unit

**DOI:** 10.1177/19375867221137099

**Published:** 2022-11-16

**Authors:** Beth Murray-Davis, Lindsay N. Grenier, Rebecca A. Plett, Cristina A. Mattison, Maisha Ahmed, Anne M. Malott, Carol Cameron, Eileen K. Hutton, Elizabeth K. Darling

**Affiliations:** 1 McMaster Midwifery Research Center, Department of Obstetrics and Gynecology, McMaster University, Hamilton, Ontario, Canada; 2 Department of Anthropology, McMaster University, McMaster University, Hamilton, Ontario, Canada; 3 Markham Stouffville Alongside Midwifery Unit, McMaster Midwifery Research Center

**Keywords:** birth unit design, midwife-led unit, architecture, maternity care, evidence-based design

## Abstract

**Background:**

Canada’s first alongside midwifery unit (AMU) was intentionally informed by evidence-based birth environment design principals, building on the growing evidence that the built environment can shape experiences, satisfaction, and birth outcomes.

**Objectives:**

To assess the impact of the built environment of the AMU for both service users and midwives. This study aimed to explore the meanings that individuals attribute to the built environment and how the built environment impacted people’s experiences.

**Methods:**

We conducted a mixed-methods study using a grounded theory methodology for data collection and analysis. Our research question and data collection tools were underpinned by a sociospatial conceptual approach. All midwives and all those who received midwifery care at the unit were eligible to participate. Data were collected through a structured online survey, interviews, and focus group.

**Results:**

Fifty-nine participants completed the survey, and interviews or focus group were completed with 28 service users and 14 midwives. Our findings demonstrate high levels of satisfaction with the birth environment. We developed a theoretical model, where “making space” for midwifery in the hospital contributed to positive birth experiences and overall satisfaction with the built environment. The core elements of this model include creating domestic space in an institutional setting, shifting the technological approach, and shared ownership of the unit.

**Conclusions:**

Our model for creating, shifting, and sharing as a way to make space for midwifery can serve as a template for how intentional design can be used to promote favorable outcomes and user satisfaction.

Canada’s first alongside midwifery unit (AMU) opened in Markham Stouffville Hospital, Ontario, in July 2018. The AMU is a physically separate birthing unit governed and staffed by midwives. The unit is in close proximity to the labor and delivery (L&D) unit, so that in the event of an emergency, patients can be easily moved. For more detail on the setup and function of the AMU, see Cameron et al. (2019). The design of the AMU was informed by existing evidence on the impact of the built environment on birth outcomes (Breedlove & Rathbun, 2019; Hodnett et al., 2009; Jenkinson et al., 2014; Lawrence et al., 2013; Newburn & Singh, 2003) and through the use of an evidence-based guide, the Birth Unit Design Spatial Evaluation Tool (BUDSET; Foureur et al., 2010; Jenkinson et al., 2014). The BUDSET is based on a set of birth environment design principles and is used to measure the optimality of birth settings (Foureur et al., 2010). The built environment refers to surroundings created for humans, by humans, that provide the setting for human activities and includes everything from landscapes to infrastructure to cities themselves (University of Windsor, 2021). The AMU’s built environment encompasses the physical rooms as well as the architecture, décor, tools, and amenities available, including the labor rooms, bathrooms, and common spaces.

Compared to conventional hospital units, the AMU was purposefully designed aesthetically as a “home-like” environment and was equipped to provide comprehensive intrapartum care. While we describe the AMU as “home-like” and much of the literature describes creating domestic birth spaces, we explicitly recognize that the concept of “home” is not universal. It is important to deconstruct this term which is pervasive in the research. In general, “home-like” is used to imply a place of safety, security, autonomy, and domesticity; however, this may not be true for all childbearing people, and for some, home may be an unsafe place (Fannin, 2003; Michie, 1998). The intention underlying the terminology is that the space is less institutional, more personal, and more familiar (Nilsson et al., 2020).

## Review of the Literature

The physical environment significantly influences service user’s feelings of safety, relaxation, sense of comfort, well-being, control, stress levels, satisfaction, and outcomes during birth (Newburn & Singh, 2003; Rudman et al., 2007; Setola et al., 2019). Further, these experiences, satisfaction, and outcomes can be improved through the incorporation of domestic features (Foureur et al., 2010; Hammond, Homer, et al., 2014; Mondy et al., 2016; Newburn & Singh, 2003; Nilsson et al., 2020; Rudman et al., 2007). For example, flexible birth rooms that give people the freedom to ambulate and give birth in upright positions can result in fewer epidurals, shorter labors, and fewer caesarean sections and are associated with fewer babies requiring admission to intensive care (Jenkinson et al., 2014; Lawrence et al., 2013). The tools and furniture in birth rooms that allow free positioning throughout labor and birth contribute to improved outcomes by fostering relaxation and supporting physiologic birth processes (Hammond et al., 2017; Igarashi et al., 2014; Jenkinson et al., 2014; Lawrence et al., 2013; Nilsson et al., 2020).

For healthcare providers, improving workplace design in healthcare settings has been shown to reduce stress, enhance safety, productivity, job satisfaction, and quality of care. This includes attention to the layout and configuration of the unit and birth room, the flexibility of the space, light and color, visual and sensory elements, a social room for healthcare providers, and incorporating elements of nature (Setola et al., 2019; Ulrich et al., 2008).

The design of the birth environment has been shown to influence job satisfaction and the way healthcare professionals provide care, which in turn may also impact birth outcomes (Hammond, Foureur, et al., 2014; Hammond, Homer, et al., 2014; Ulrich et al., 2008). Traditional hospital-based birthing units have been identified as sometimes being a challenging setting for midwives, as the environment is perceived to be overly medicalized and not designed to facilitate or support care that optimizes physiologic birth and may even directly hinder it (Foureur et al., 2010; Hammond et al., 2013; Hammond, Homer, et al., 2014). An improved birth unit design, therefore, must support the physical, functional, and psychological needs of midwives to support the provision of quality care and meet the needs of the person giving birth (Hammond, Homer, et al., 2014).

The purpose of this study was to assess the impact of the built environment of the first Canadian AMU for midwifery service users, along with the midwives who work there. Our specific objectives were to explore the meanings that individuals attribute to the built environment and to explore how this influences satisfaction with birth space.

## Method

### Study Design

We conducted an explanatory, sequential mixed methods using a grounded theory methodology. This study was one component of a concurrent, multiphase, mixed-methods evaluation of the AMU, involving both midwives and midwifery service users. Ethics approval was granted by both the Markham Stouffville Hospital (Protocol #78-1808) and Hamilton Integrated Research Ethics Boards (Protocol # 5050 and 5056).

### Sociospatial Conceptual Approach

Our study was informed by a sociospatial conceptual view of the birth environment. From this perspective, the birth environment is defined as not only a physical space but also a place that has meaning and value invested within it by the people who interact with it. The birth environment is shaped by what is physically in the room, such as the design elements, equipment, and the architecture, but also by broader sociocultural expectations, norms, and influences, which are constructed in part by the thoughts, feelings, and responses of the people who use and interact with the environment (Hammond et al., 2013). We used this approach in developing our research questions and data collection tools.

### Recruitment and Inclusion Criteria

**Figure 1.**
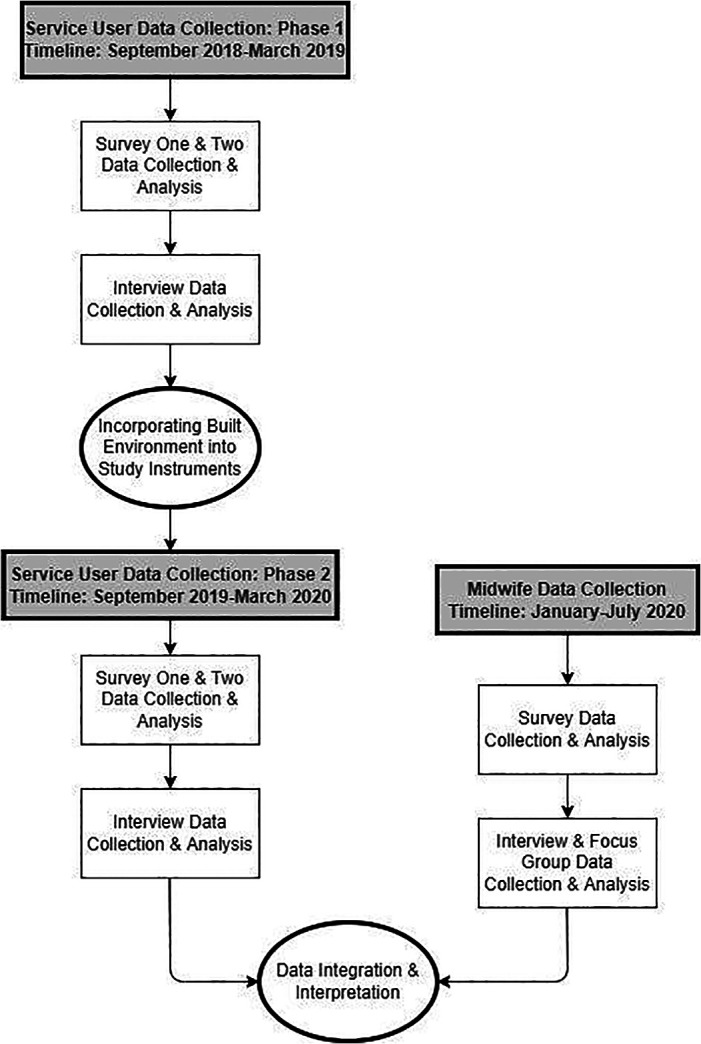
Mixed-methods research design.

Our study population included midwifery service users and midwives. Everyone admitted to the AMU, including individuals whose care was later transferred to an obstetrician on the L&D unit during the intrapartum period, was eligible to participate in the study (Figure 1). We recruited these participants during two time periods, September 2018–March 2019 (Phase 1) and September 2018–March 2020 (Phase 2). They completed a consent to contact prior to hospital discharge and a survey link was emailed 2-weeks later. The survey included an invitation to participate in a follow-up interview conducted by telephone. All midwives who attended births at the AMU from January to July 2020 were eligible to participate and were recruited via email.

### Data Collection

Midwifery service users completed an online survey, followed by an individual interview. Midwives participated in either an individual interview or a focus group. The survey and interview questions for all participants, developed by our research team, explored experiences of the built environment of the AMU, including how they used the space, their satisfaction, and evaluation of the equipment available to them. Individual interviews, completed via telephone, and the focus group, conducted in person, were conducted by an experienced researcher and audio recorded. We obtained informed consent from all individuals prior to their participation in all surveys, interviews, and focus groups.

In keeping with grounded theory approaches, we conducted data analysis concurrently with data collection to facilitate the iterative process of constant comparison (Glaser & Strauss, 1967). Decisions to refine the interview guide were made during peer debriefing meetings and agreed on by the research team. We revised the client Phase 2 survey and interview guide after interim data analysis revealed that the physical environment was an important factor shaping birth experiences. As a result, the Phase 2 survey and the interview guide were modified to include more explicit questions about the impact of the physical environment. We continued recruitment until data saturation was reached and no new data was emerging from the interviews (Sandelowski, 1995).

### Data Analysis

The data were analyzed sequentially. First, we analyzed the quantitative survey data. Descriptive statistics were used to present the survey data using Microsoft Excel. The Likert scales were collapsed into a three-point scale (Agree, Neutral, and Disagree) and summarized into percentage proportions. Next, we used grounded theory to analyze our qualitative data. Specifically, data collected from interviews were transcribed verbatim, read line-by-line, and open coded by three researchers. To ensure reliability between coders, codes were reviewed as a team to ensure agreement. We created categories by identifying relationships between related open codes; next, we clustered categories together to form themes (Charmaz, 2003). These themes were used to develop a theoretical model. Triangulation of multiple data sources, methods, and investigators was used to ensure reliability and validity of our findings (Carter et al., 2014).

## Results

Fifty-nine service users completed the survey. Twenty-eight services users and 11 midwives participated in interviews and three midwives participated in one focus group (Figure 1).

The majority of midwifery service-user participants had postsecondary education, earned a total household income above US$100,000 per year, and were similar in age (Table 1). Participants identified as East Asian (8.5%), South Asian (15.3%), White-European (13.6%), and White-North American (47.5%). Due to the small number of midwifery participants, we did not collect detailed demographic data to preserve their anonymity; however, most of midwives had worked 3–10 years in healthcare (40.9%) and worked at that hospital between 3 and 10 years (45.5%; Table 2).

**Table 1.** Demographic Characteristics of Survey Respondents.

Characteristics	Phase 1	Phase 2	Total
	*n* (%; *n* = 63)	*n* (%; *n* = 59)	*n* (%; *n* = 122)
Gender			
Female	62 (98.4)	63 (100)	121 (99.2)
Age			
20–24	2 (3.2)	2 (3.4)	4 (3.3)
25–29	16 (25.4)	15 (25.4)	31 (25.4)
30–34	31 (49.2)	21 (35.6)	52 (42.6)
35 or older	14 (22.2)	21 (35.6)	35 (28.7)
Parity			
Primiparous	36 (57.1)	25 (43.1)	61 (49.6)
Multiparous	27 (42.9)	33 (56.9)	62 (50.4)
Education			
High school diploma or equivalent	5 (7.9)	4 (6.8)	9 (7.4)
Postsecondary nonuniversity (e.g., apprenticeship/trades, college/nonuniversity certificate/diploma)	14 (22.2)	15 (25.4)	29 (23.8)
University certificate/diploma	44 (69.8)	39 (66.1)	83 (68.0)
Prefer not to answer	0 (0)	1 (1.7)	1 (0.8)
Income			
Less than US$20,000	0 (0)	2 (3.4)	2 (1.6)
US$20,000–US$49,99	5 (7.9)	2 (3.4)	7 (5.7)
US$50,000–US$99,999	31 (49.2)	19 (32.2)	50 (41.0)
Over US$100,000	27 (42.9)	29 (49.2)	56 (45.9)
Prefer not to answer	0 (0)	7 (11.9)	7 (5.7)
Ethnic identity ^a^			
Asian-East	N/A	5 (8.5)	—
Asian-South	N/A	9 (15.3)	—
Asian-South East	N/A	3 (5.1)	—
Black-African	N/A	1 (1.7)	—
Black-Caribbean	N/A	2 (3.4)	—
Latin American	N/A	1 (1.7)	—
Middle Eastern	N/A	1 (1.7)	—
White-European	N/A	8 (13.6)	—
White-North American	N/A	28 (47.5)	—
Mixed heritage	N/A	1 (1.7)	—

*Note.* Percentages may not add up due to rounding.

^a^ Ethnic Identity was not collected in Phase 1 of data collection. No participants in Phase 2 were of Black-North American, First Nations, Indian-Caribbean, Indigenous/Aboriginal (not included elsewhere), Inuit, Metis, or Other heritage.

**Table 2.** Demographics of Midwife Participants.

Midwife Demographics	*n* (%; *n* = 22)
Time in healthcare	
0–2 years	4 (18.2)
3–10 years	9 (40.9)
11–15 years	2 (9.1)
Over 15 years	7 (31.8)
Time at MSH	
0–2 years	6 (27.3)
3–10 years	10 (45.5)
11–15 years	2 (9.1)
Over 15 years	4 (18.2)

### Quantitative Findings

**Figure 2.**
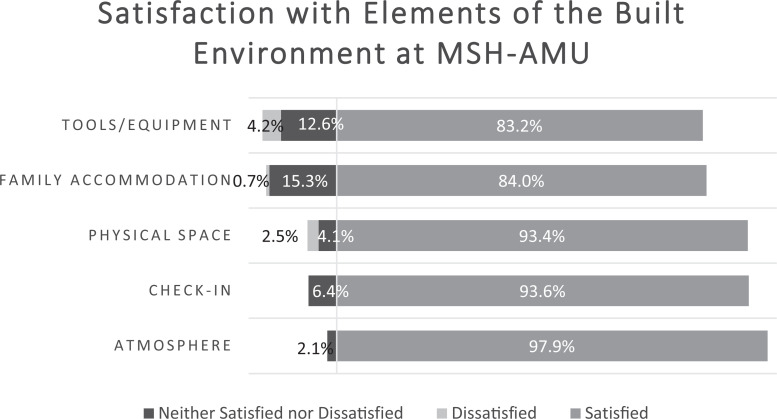
Service user satisfaction with elements of the built environment at the MSH-AMU. Atmosphere: levels of privacy, noise, and calmness. Physical space: walking space, birthing room, and general unit. Tools and equipment: satisfaction with tools available and murphy bed. Family accommodation: space and furniture available to family members and the family lounge/kitchen.

**Figure 3.**
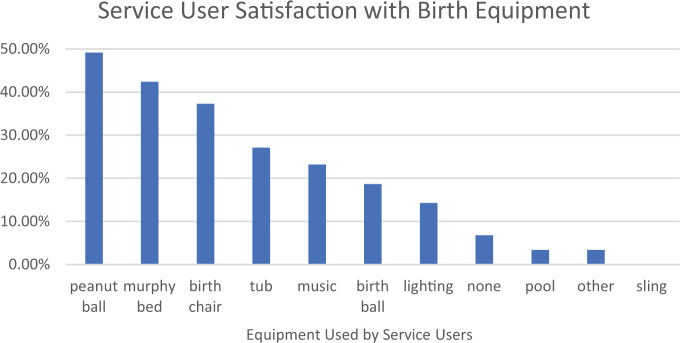
Service user satisfaction with birthing equipment utilized at the MSH-AMU.

The survey findings indicated that service users were highly satisfied with their experiences in the AMU and with elements of the built environment. Components of their experiences that were rated as highly satisfying included: the check-in/welcome experience (93.6%), atmosphere (97.9%), physical space (93.4%), the tools and equipment available (83.2%), and the accommodations made for family members (84.0%; Figure 2). In terms of birth equipment, the peanut ball (49.1%), murphy bed (42.4%), and birth chair (37.3%) were used most frequently, while the birth pool (3.4%) and sling (0%) were least frequently used (Figure 3).

### Qualitative Findings

**Figure 4.**
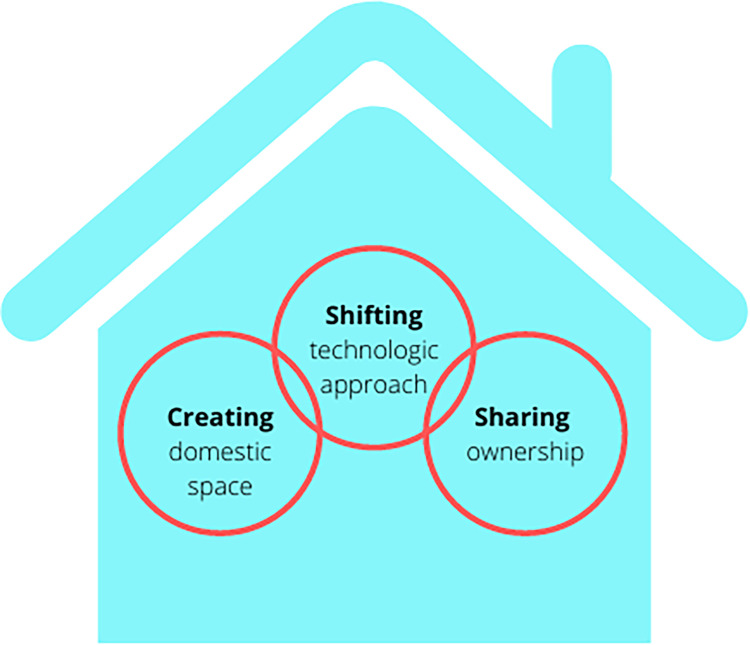
The creating, shifting, sharing theoretical model demonstrating the three elements that contribute to making space for midwifery-led care within the hospital.

**Figure 5.**
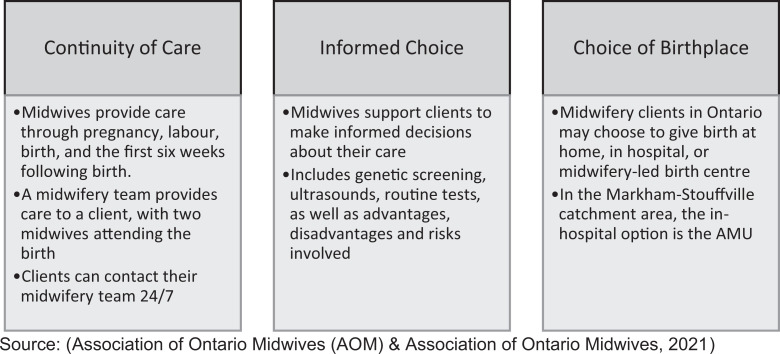
Ontario midwifery philosophies of care. *Source.* Association of Ontario Midwives (2021).

Our interview findings confirmed and expanded on the survey findings and supported the development of a theoretical model, whereby “making space” for midwifery in the hospital contributed to positive experiences and overall satisfaction with the built environment of the AMU. “Making space” is accomplished through three crucial steps: creating a domestic space in an institutional setting, shifting the technological approach, and sharing ownership of the space (Figure 4). We use “making space” both literally, as the AMU is a separate physical space designed and built in the hospital, and figuratively, as midwives and their service users were given the space, power, and freedom to labor and birth independently according to midwifery philosophies of care (Figure 5). We review each theme below and participant quotes have been organized by theme in Table 3.

**Table 3.** Respondent Quotes.

Themes	Respondent Quotes
Creating a domestic space in an institutional setting	Service user
	I liked the peacefulness of it. It felt very relaxed and Zen…. compared to a regular hospital unit where you have four people in a triage room, and you have people screaming, and people all around you, this was much more secluded, much quieter, which really made me feel like I could relax during labour, which I really important to me
	It didn’t feel like a typical hospital room by any means, with the beautiful murals, and everything, it just kind of provided a different atmosphere, and I also loved the option for calm hallways where we could walk as I was labouring, and read the motivational quotes on the wall
	It was just enough space. It wasn’t too crowded. It allowed me to bring my belongings and allowed me to walk around the room without having to feel like everyone was around me that it was too close, so I liked our actual room, and it was very private and just the perfect size.
	I am so happy I went through that method, because you really have the comfort and relaxation as if you were in a home setting, but still have the expertise and the team available to you to make everything go smoothly. ***I am so happy I went through that method, because you really have the comfort and relaxation as if you were in a home setting, but still have the expertise and the team available to you to make everything go smoothly.***
	It was a really good set up for my partner. The rooms have comfy couches. They have lounge chairs. There’s a kitchen. Partners can be looked after and feel part of the process too, I think, as much as they can
	They had lots of space so that my family could be in the room with me, which was extremely important to me
	Midwife
	It seems quiet. Even when we’re really, really busy, it never feels as loud or as busy as it did over on labour and delivery
	MW-B: It’s just less cluttered, too, with less people milling around. There’s less bothering them, so it feels just like an organized space so you could come in and be ready to do what you need to do MW-C: and you go over [to L&D], and It’s almost like the walls are coming in on you and I think, too, because There’s so many more people in the same space, whereas you come over here and you might only have one or two people on the entire unit
	I think it’s the space itself is really helpful to labouring women, because the attempt was to make it more of a homelike experience, because often when they’re leaving home and then coming into a new environment, it can be a bit stressful, which can shut labour down a little bit, slow it down. So the attempt to mimic a homey type atmosphere, I think, is encouraging to the labour progress itself
Shifting the technological approach	Service user
	The birthing rooms were great. The Jacuzzi tubs were great. I laboured in that for a bit. They had bars along the wall to help to squat or whatever and whatnot during contractions. The birthing stool was awesome, the Murphy bed. No, overall, the environment was great…. This is also why I went the midwifery route is because you can labour in whatever way you want, right? And there were just so many options, right? I went from the bathroom where there was a Jacuzzi, like the jets. The bars on the wall, I could do it on the bed. I was on all fours on the ground. I was in the birthing stool. I was lying flat on the floor. There was tons of space around me and I think that allowed me to kind of like be in whatever position I wanted, which is exactly what I needed
	The ability to move around. It was really accommodating to being in labour I felt. Just having…the space to really labour in different ways was fantastic. It made it feel not closed in or anything or rushed. It just kind of felt like…the space was there to use as we needed it, which was great. It felt like our space
	I think having it being able to kept away gave us that extra space for me to pace and just even allow us labouring in the one spot, just that openness helped
	Midwife
	Yeah, it’s a hospital room. Don’t get me wrong. There’s oxygen on the wall, there’s all of that, but there isn’t a lot of clinical equipment. So there isn’t that anxiety that comes right away, I’m in a hospital room with a bed and a warmer and all of those things. It’s all tucked away. It’s all hidden, it’s covered
	There’s just so many options that the women and the clients, they’re all up to try anything. And it’s almost like the midwives will have a bigger toolbox full of tools that they can use. I mean sterile water, the TENS machine. They can bring the mom and put her in the bathtub or they can do a water birth if they want. There’s so many more tools that we can use
	It felt like we didn’t really use the entire room before on the other unit, like it was the same space and everything, but I felt like we used half of the room…whereas now it’s kind of like no, just use the whole thing. Like I find that even, I get them to sit on the chairs that were designated for us and like just anywhere. Live your life anywhere in this room, the whole thing is yours. I see more clients pulling out their personal belongings…pictures and things to make it feel a little bit homier for themselves
	[O]ne of the outcomes of the physical space, I know has been to encourage more physiological positioning in labour, more upright positioning in births and that is definitely something the way that the physicality of each room is laid out, that it really enables that. Anybody’s who has sort of worked in the birth environment will know that if you bring somebody labouring into a room and there’s a bed there, it’s very easy for that person to just climb into the bed and lay there. But we know that that’s not the best place to labour. So in our rooms, the bed is not immediately visible unless this client is requesting an epidural and we know that that’s in their birth plan and that’s what they’re going to do when they become active in labour. And that’s definitely something that I’ve seen as well. It’s very clear that like clients are more physiological in their labouring rather than sort of gravitating towards a flat surface and lying down
	For the most part, the Murphy beds are tucked away. They’re there when women need to relax after birth. Or if they need a little rest during labour, they can lie on their side on this Murphy bed that pulls down. But ambulation, upright posture, in a Rebozo sling type device, in the water, all of these approaches to supporting labouring people are well supported by the research literature to help facilitate normal physiologic birth
Shared ownership of the space	Service user
	If there’s anything that makes it feel more homey. I brought in a bouquet of flowers, because I love flowers and candles. I had LED candles in there with me just for additional ambient light, because I like low lights and warm lighting, and that always makes me feel more at home
	It just kind of made it feel like we had ownership…over our birth experience, because we had this space that kind of just felt like our own that we could use to walk around and made it like—and get into different positions that were helpful to labour, while also just kind of keeping calm as best as possible as well. ***It just kind of made it feel like we had ownership…over our birth experience, because we had this space that kind of just felt like our own that we could use to walk around and made it like—and get into different positions that were helpful to labour, while also just kind of keeping calm as best as possible as well.***
	It was fantastic. There were a couple of chairs for him to sit in. He felt welcome. There was the family area for him to use, the toaster, whatever it was, fridge, and the ice machine. He felt pretty comfortable. I feel like he felt the space was also his
	Midwife
	It feels like there’s a great degree of ownership both from the hospitalists there and the community midwives who work there. And there’s a different feeling when you come onto the floor as opposed to just walking around labour and delivery…just having a sense of like this is our place and it reflects our philosophy of care
	[We] have a pantry and the working environment for the midwives, there’s a team room, there’s a kitchen, there’s space for the hospitalists to work. So, to me, that is very reinforcing of validating everyone’s job and everyone’s work there, and making sure that they have space that they call their own
	Having the ability to change the colour of the lighting and putting on their music, their playlists, their baby playlists, they really like…they feel safe, they feel comfortable. They’ve made it their own little environment and I think that helps them stay calm, stay relaxed. They can have more interactions with their partners. We let visitors come in and I think that really helps them feel supported as well. And all those things just contribute to progress and for their labour to go well, for them to have a positive experience and a good experience
	They have no idea what a birth stool is. They’ve probably never heard about it. They don’t know what it’s useful for. They don’t know that it’s going to help them. A lot of them don’t even know about like a birth ball is helpful
	I find having the couches out of there makes the partner be more involved. And it’s just a chair that’s not super inviting and then having us be in the whole space, it’s like you have to be involved or otherwise you’re just in the corner. Yeah, they’re really engaged and they like all the stuff. They seem to like all the things we have

### Creating a Domestic Space in an Institutional Setting

The AMU was designed to look and feel cozy, relaxing, comfortable, and intentionally different from the traditional hospital environment, which participants described as “cold and sterile.” Despite being located within the hospital, the atmosphere, aesthetics, spaciousness, and the ways it supported family members all contributed to the AMU feeling more domestic than institutional. The atmosphere was described by midwives as being quiet, calm, spa-like, and very welcoming to families.

**Figure 6.**
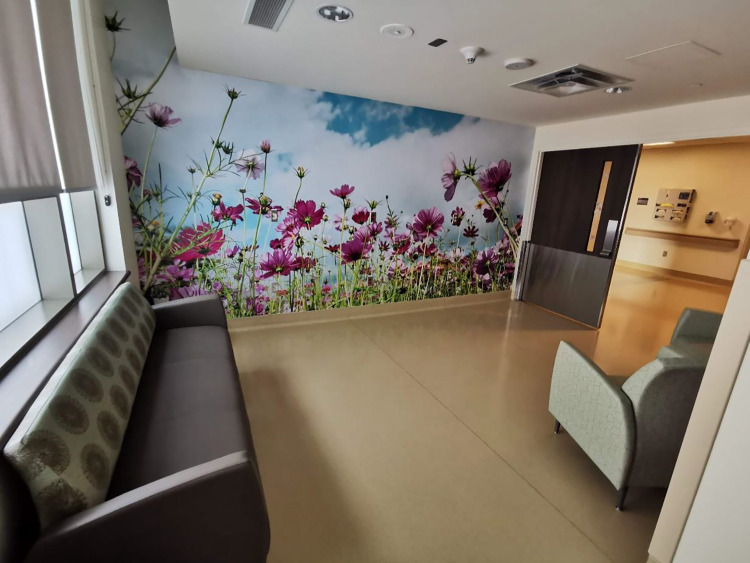
Natural light and images of nature in birth room.

Service users chose similar words to describe the atmosphere and went further to articulate that it felt private and intimate and noted the atmosphere was very different from a hospital ward. Multiparous people who had previously given birth on the traditional labor unit noted that the AMU was calmer in comparison. The AMU aesthetics included soft wall colors, nature murals, artwork, birth affirmations, and soft furniture (Figure 6). Service users appreciated that the space’s design and aesthetic felt very intentional to support the philosophies of midwifery care. They described how the ambiance, including music and lighting customization, contributed to their comfort and relaxation in the space, feeling strongly that their surroundings impacted their mood and birth experience.

**Figure 7.**
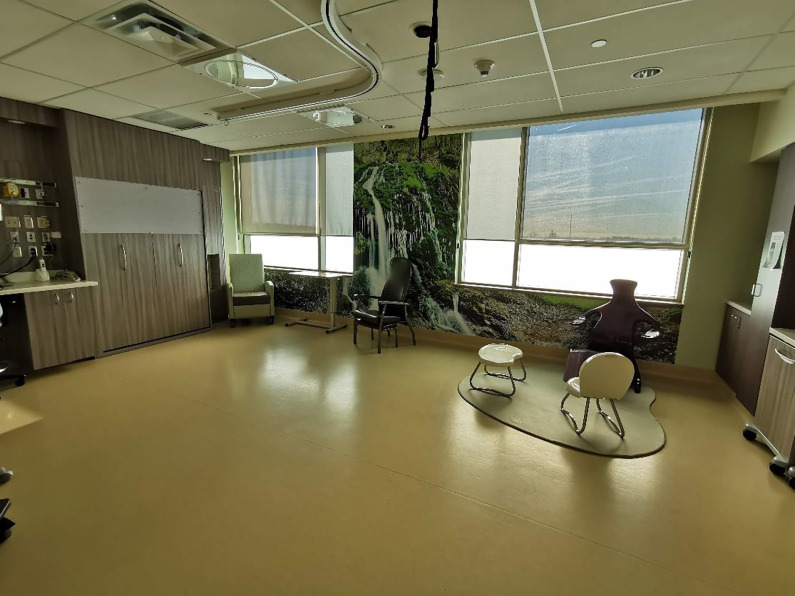
Spacious birthing room.

Both service users and midwives enjoyed the spaciousness of the AMU and birthing rooms. The AMU was less crowded and busy than the traditional labor unit due to fewer people, fewer visible supplies, and medical equipment, resulting in more open spaces (Figure 7). The midwives noted that the birthing rooms were free from “clutter”—such as the traditional hospital bed, pumps and preprimed intravenous sets, and most obstetric equipment, making the room feel more spacious. The service users appreciated the sense of space they felt in their room.

A major design element of the AMU was to create an environment that felt more like a domestic space than a hospital. Service users commonly described the AMU as “the best of both worlds,” meaning the best of homebirth and hospital birth combined. This referred to the ability to have a home-like environment while still being in the hospital, with quick, easy access to obstetrical care if needed, unlike at a planned homebirth. The space also accommodated and supported partners and families through the family lounge and kitchen, comfortable furniture, and the birthing room being large enough to comfortably accommodate their presence. Families also expressed feeling welcome in the space by AMU staff, noting that the space was set up to support their involvement in the birthing experience.

Overall, the ambiance, aesthetics, and family-orientation created a domestic space that contributed to feelings of comfort and relaxation.

### Shifting the Technological Approach

**Figure 8.**
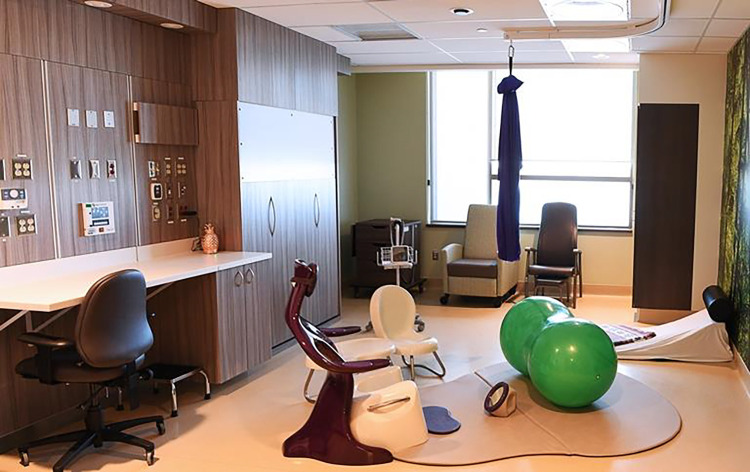
Variety of tools available in birthing rooms at alongside midwifery unit.

The design of the AMU moved away from the biomedical and technological focus of childbirth characterized by a reliance on technology. This shift occurred by removing from the birth space much of the technology that was not routinely used. The primary motivation for this was to decrease access to unnecessary equipment and therefore demonstrate respect for judicious rather than routine use of technology. Put simply, if the continuous fetal heart rate monitor is present in the room, it will be used. However, this removal of equipment had two consequences. First, it reduced the amount of unnecessary equipment, which created more open space for people to move about and labor freely. Second, it allowed for the introduction of nontechnology-based labor and birth tools and equipment. For example, since the traditional equipments, such as the fetal heart monitor, obstetric bed, and neonatal warmer. were brought in only when needed, items to support physiologic birth, such as bars, slings, mats, and birth stools, were prioritized and visible. On the traditional labor ward, these tools had previously not been available, not accessible due to space limitations. By comparison, in the purpose-built AMU, these tools and equipment were at the midwife’s fingertips and promoted their use to support physiologic birth (Figure 8). The tools were conveniently located, and they had more of these tools at their disposal than ever before.

Service users enjoyed and appreciated the variety of resources available since it gave them more opportunities to find the right tool or position while coping with contractions. However, some service users felt they needed better education about the tools, how to use them, how they were beneficial in labor, and at what stages in labor they were helpful. Without this information, clients often refrained from using the equipment at all. For example, some service users reported not knowing about the ability to control the lighting and music in the room.

Overall, both service users and midwives reported that removing and modifying the technology and equipment in the birthing room created a space that was flexible and usable. Midwives had the ability to remove, move, or add equipment as necessary to accommodate the needs of each individual. The open space facilitated mobility, freedom of movement, and the ability to use more of the space.

**Figure 9.**
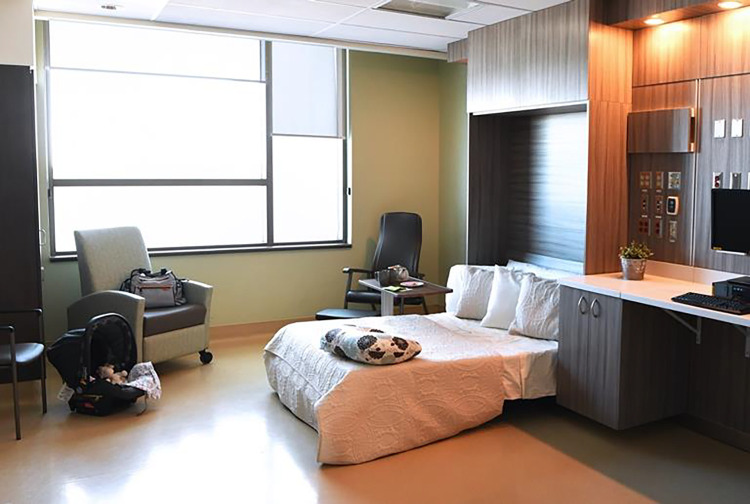
Double murphy bed.

A unique design feature of the AMU birth spaces was the replacement of traditional hospital beds with a queen-sized murphy bed that was folded down from the wall only when in use (Figure 9). This shifted the focus away from the bed being the center of the room. The bed was hidden until needed, which provided more space in the room for the client to use, be mobile in, and have family present. The midwives described that ideally the bed would only be used for postpartum recovery, but many service users still expected they would labor and give birth on a bed and desired this experience. Feedback from service users indicated that they appreciated the murphy bed particularly in the postpartum time when they could relax and bond with their baby. They also liked that it was big enough to fit their partner. Not having the bed directly available really reinforced movement and encouraged service users to try other tools and equipment. However, while there were benefits of this style of bed, it was articulated as not being ideal for perineal suturing due to its low height, which placed ergonomic strain on midwives.

### Sharing Ownership of the Space

We found that all users of the space, service users, midwives, and family described a sense of individual ownership over the space. For midwives, this stemmed from the space being solely for midwifery care and governed and run by midwives. The governance of this space was an important aspect of ownership, as midwives were able to create their own care standards based on evidence-based midwifery approaches to care. Midwives recounted how this facilitated a sense of power and autonomy, knowing that the space was designed specifically to support their philosophy of care and validated their work. Service users supported this sentiment, valuing a space that was designed to promote the type of care and birth philosophies they were seeking when choosing midwifery care. Features like the staff room and meeting rooms gave midwives a sense of belonging and camaraderie that had been missing since they had never previously had dedicated spaces in the hospital.

Those birthing on the unit demonstrated multidimensional ownership of the space, by taking control of their physical surroundings and their birth experience. In the birthing room, they had flexibility to modify the space, including the equipment, labor tools, and furniture. They also had the ability to personalize the lighting and music and were encouraged to bring personal items to make the space more comfortable, such as LED candles, pictures, and curated music playlists. The birthing room was described as a space that became their own, almost an extension of their home. The built environment also facilitated greater ownership over the birth experience. This was achieved by using a variety of birthing tools, hydrotherapy, and movement into various positions for laboring and pushing, and ambulating in the room and in the hallways. Midwifery service users expressed feeling a sense of control over their experience as a result of having more options. The midwives noted that ownership and customization of the space contributed to relaxation, improved outcomes, and an overall better birth experiences for service users.

Finally, family members also shared in this sense of ownership since they were able to be an integral part of the birth experience. All participants expressed appreciation that families had enough space in the birthing room to settle in and be comfortable, without feeling crowded and that spaces such as the lounge and kitchen accommodated families. Additionally, the midwives believed that partners were more involved in labor support on account of the increased space and room for movement.

## Discussion

This study, underpinned by a sociospatial conceptual approach, used grounded theory to explore the impact of the built environment of Canada’s first AMU from the perspective of both midwives and service users. The findings revealed that the intentional design elements of the unit facilitated positive experiences and contributed to greater satisfaction with the birth experience. We attribute these positive experiences to three key ways of “making space” for midwifery-led birth—creating a domestic space in an institutional setting, shifting the technological approach, and sharing ownership of the AMU (Figure 5). These findings contribute to the growing literature advocating for the benefits of evidence-based birth unit design (Berg et al., 2019). Framing these findings within the sociospatial approach, it is clear that the experiences of midwives and service users were not due to the space alone, but how the space was used, which was co-created by everyone who entered the unit, and was underpinned by midwifery philosophies (Figure 5).

Our results support previous research, which emphasized the importance of ownership and control in the birthspace (Igarashi et al., 2014; Mondy et al., 2016; Setola et al., 2019). The birthing rooms and family lounge contributed to families feeling welcomed and involved in the birthing process (Mondy et al., 2016; Nielsen & Overgaard, 2020; Setola et al., 2019). The unit was described as “the best of both worlds,” a similar finding to that of Kuliukas et al. (2016) in a co-located birth center in Australia (Kuliukas et al., 2016). These findings support offering a third option to give birth, as neither home nor traditional hospital may be the optimal fit.

The flexibility of the space was a key component to participants’ satisfaction, making it easier for midwives to fully promote physiological birth and giving birthing people ownership and options (Hammond, Homer, et al., 2014; Setola et al., 2019). It is important that this flexibility was recognized and utilized by both care provider and service users since the birthing environment must meet the needs of everyone in the space (Dana & Wambach, 2003; Setola et al., 2019; Skogström et al., 2022). In traditional hospital labor wards, midwives often attempted to modify the physical environment to try and make the space more domestic or to facilitate normal birth (Bourgeault et al., 2012; Davis & Homer, 2016; Igarashi et al., 2014) but were constrained by what the space would allow (Mondy et al., 2016). Our findings are in line with those of Hammond et al. (2013), demonstrating that the ways in which midwives use the birth space is impactful, as midwives were more effective at promoting physiologic, low risk birth when working in an environment that supports their professional needs (Hammond et al., 2013).

Our findings stress the impact and importance of midwives feeling a sense of ownership and belonging in the birthspace. Previous findings from Bourgeault (2012) found that midwives felt like visitors in labor wards and guests at homebirths and described “borrowing space” that explicitly did not belong to them (Bourgeault et al., 2012). This newfound sense of ownership, belonging and control described by midwives on the unit is an important finding and warrants further research to explore how this impacts practitioner confidence, professional identity, and job satisfaction.

As now neither a visitor in clients’ homes, nor guest in the labor ward, midwives working on the AMU exist in a liminal space, which Brown et al. (2017) described as phenomena that do not fit into normal categories but fall “betwixt and between.” The unit is neither home, nor hospital, yet has overlapping elements with both. The liminality of the unit is potentially why the space is so effective for clients and midwives, as its flexibility allows the space to be used in whatever ways are needed by clients or their caregivers. The space can become whatever is desired, providing both parties a sufficiently strong internal locus of control to feel a sense of ownership of the space. Its character as neither home nor institution alleviates the risk narrative of home birth, and the flexibility of the space allows for the tenet of choice to be made manifest.

### Implications for Practice

As Canada’s first AMU, this analysis is critical for identifying factors that should be considered when designing future birth spaces to optimize the environment for all. Our model for creating, shifting, and sharing as a way to make space for midwifery approaches can serve as a template for other centers looking at intentional design to promote favorable outcomes and user satisfaction.

This model of care and the intentional design of the birth environment may be an important component to midwifery retention, which is an ongoing problem globally. In a recent study in Western Canada, one third of midwives surveyed had seriously considered leaving the profession (Geraghty et al., 2019). The literature thus far has not specifically addressed what impact the physical environment and its association with autonomy may contribute to midwife job satisfaction and attrition. However, our findings suggest that this type of model may help contribute to better midwifery retention through both cognitive and physical benefits. Decreased stress levels, greater satisfaction at work, and the shift away from hierarchical power structures may address common sources of burnout (Bloxsome et al., 2019; Geraghty et al., 2019; Stoll & Gallagher, 2019).

There are further implications to using “home-like” as a design goal. From an equity lens, there may be a benefit to the unit having a stereotypical “home-like” aesthetic, as it provides an alternative to homebirth for those who don’t have a home-space that provides the same safety, comfort, ambiance, and spaciousness as the AMU.

Our mixed-methods approach strengthened the study as we were able to confirm our findings through data triangulation. Limitations of the study stem from the fact that we did not collect data from patients with other care providers at the same hospital, nor did we investigate midwifery service user’s experiences in the traditional labor ward prior to the opening of the midwifery unit. Also, our findings may not be generalizable to other communities due to the specific demographics of the midwifery population who participated.

## Conclusion

The built environment of AMU resulted in positive experiences and satisfaction among all participants. The domestic environment was relaxing and comfortable for those in labor, while still maintaining proximity to emergency care. This purpose-built space designed with equipment and resources to support midwife-led care encouraged flexibility and choice for those giving birth. Finally, the built environment led to a greater sense of ownership for all those giving and receiving care. This went beyond the space and how it is designed, to include how it was used and co-created by midwives, service users, and their families.

## Implications for Practice

- To optimize the birth environment for all, institutional birth settings should consider incorporating domestic elements and demedicalizing the space to promote midwifery approaches to care which support physiologic birth.
- Midwifery ownership and self-governance contribute to how the space is used and co-created with service users. This agency and ownership foster a sense of belonging, which is an important contributor to healthcare provider job satisfaction.
- A positive built environment for the birth setting may have a meaningful impact on addressing attrition and retention in the midwifery profession through decreased stress and increased job satisfaction.
- Optimizing the birth environment can improve childbirth satisfaction among women and birthing people and their families. But service users need to be informed about how to optimally interact and make use of the space during the antenatal period.
